# Targeting Microglia and Macrophages: A Potential Treatment Strategy for Multiple Sclerosis

**DOI:** 10.3389/fphar.2019.00286

**Published:** 2019-03-22

**Authors:** Jiaying Wang, Jiajia Wang, Jincheng Wang, Bo Yang, Qinjie Weng, Qiaojun He

**Affiliations:** ^1^Zhejiang Province Key Laboratory of Anti-Cancer Drug Research, College of Pharmaceutical Sciences, Zhejiang University, Hangzhou, China; ^2^Center for Drug Safety Evaluation and Research, Zhejiang University, Hangzhou, China

**Keywords:** multiple sclerosis, microglia, macrophages, central nervous system, targeted therapy

## Abstract

Multiple sclerosis (MS) is a chronic inflammatory neurodegenerative disease of the central nervous system (CNS). The early stage is characterized by relapses and the later stage, by progressive disability. Results from experimental and clinical investigations have demonstrated that microglia and macrophages play a key part in the disease course. These cells actively initiate immune infiltration and the demyelination cascade during the early phase of the disease; however, they promote remyelination and alleviate disease in later stages. This review aims to provide a comprehensive overview of the existing knowledge regarding the neuromodulatory function of macrophages and microglia in the healthy and injured CNS, and it discusses the feasibility of harnessing microglia and macrophage physiology to treat MS. The review encourages further investigations into macrophage-targeted therapy, as well as macrophage-based drug delivery, for realizing efficient treatment strategies for MS.

## Introduction

Multiple sclerosis, a CNS autoimmune and neurodegenerative disease, affects approximately 2.5 million people worldwide, seriously diminishing their quality of life and causing a significant financial burden (Compston and Coles, 2008; Lemus et al., 2018; Thompson et al., 2018). Studies using EAE, an animal model of MS, have revealed that microglia/macrophages actively participate in the pathogenesis of EAE progression (Jiang et al., 2014). The CNS contains several resident macrophages, such as microglia and non-parenchymal macrophages, in the choroid plexus, perivascular space, and meninges, which function to maintain CNS homeostasis (Prinz et al., 2011). Alterations in CNS homeostasis lead to the recruitment of peripheral blood-derived monocytes (known as monocyte-derived macrophages) into the CNS, the activation of microglia, and the presence of foamy macrophages (Bogie et al., 2014; Kuhlmann et al., 2017; Mrdjen et al., 2018; Zéphir, 2018). This phenomenon appears to be a type of self-protective mechanism designed to eliminate abnormalities and recover a steady state.

Monocyte-derived macrophages and CNS-resident phagocytic cells can promote neuroinflammation, thereby inducing MS, but they can also play neuroprotective and anti-inflammatory roles, depending on many factors. In this review, we discuss the protective and pathogenic mechanism of microglia and macrophages in the development of MS and EAE, and propose the use of macrophages in MS therapy for repairing CNS damage.

## Microglia and Macrophages in a Healthy CNS

In a healthy CNS, microglia reside in the parenchyma, while non-parenchymal macrophages are present in boundary regions, including perivascular spaces, the meninges, and the choroid plexus. These cells have different developmental origins and serve distinct functions by specific subtypes.

### Origin of CNS Macrophages and Microglia

**FIGURE 1:**
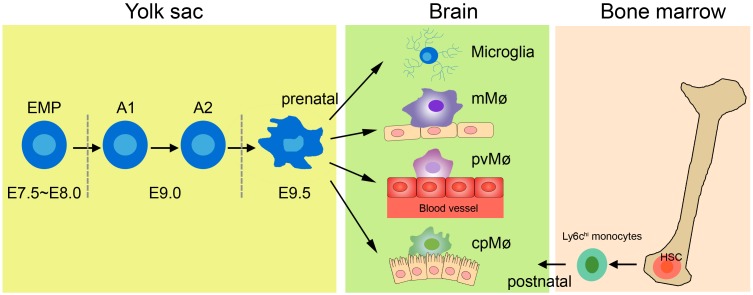
Origin of tissue macrophages in healthy CNS. Microglia and tissue-resident macrophages are derived from prenatal sources. The primitive erythromyeloid precursors (EMPs) located in the yolk sac during E7.5∼8.0 soon develop into A1 cells and finally become A2 cells. The matured A2 cells then differentiate into microglia, meningeal macrophages (mMø), perivascular macrophages (pvMø), and choroid-plexus macrophages (cpMø). However, after birth, cpMΦ originate exclusively from bone Ly6c^hi^ monocytes.

The earlier view that CNS macrophages are derived from blood-borne myeloid cells during adulthood has now been challenged, because studies using novel transgenic mouse models have shown that macrophages other than choroid plexus macrophages originate exclusively from the yolk sac (Figure 1; Gomez Perdiguero et al., 2015; Goldmann et al., 2016). The application of tamoxifen to adult *Cx3cr1*CreER:*R26*-YFP mice has further demonstrated that not only microglia but also perivascular and meningeal macrophages retain the yfp label from embryonic day (E)16.0 to 8–9 weeks of age (Goldmann et al., 2016), indicating that non-parenchymal meningeal and perivascular CNS macrophages and microglia arise entirely from embryonic precursor cells. Fate-mapping tools have helped reveal that CNS macrophages arise from the primitive c-kit^+^ erythromyeloid precursors located in the yolk sac during E7.5∼8.0. Uncommitted erythromyeloid precursors subsequently disappear at E9.0 and develop into CD45^+^CX3CR1^hi^F4/80^hi^ (A2) macrophage progenitors via immature CD45^+^CX3CR1^lo^F4/80^lo^ (A1) cells. Proliferating A2 cells then become CD11b^+^F4/80^+^ microglia and perivascular, meningeal, and choroid plexus macrophages (Bertrand et al., 2005; Kierdorf et al., 2013; Hoeffel et al., 2015; Matcovitch-Natan et al., 2016). Once established in the CNS, microglia and perivascular and meningeal macrophages persist throughout the life of the organism due to their longevity and their capacity of self-proliferation, rather than the infiltration of peripheral myeloid cells (Ajami et al., 2007, 2011). In contrast, choroid plexus macrophages mainly depend on blood-derived immigrating Ly6C^hi^ monocytes after birth (Goldmann et al., 2016). The astonishing finding of such close ontogeny between microglia and the other non-parenchymal macrophages in the CNS prompts the question of whether they can transform into one another under specific physiological conditions.

### Functions of Microglia

Microglia are widely scattered throughout the brain and spinal cord, and they come into close contact with neurons, astrocytes, and oligodendrocytes (Ransohoff and Cardona, 2010; Szepesi et al., 2018). Studies have demonstrated that microglia mediate synaptic pruning and trophic-factor production through fractalkine-CX3CR1 signaling in neurodevelopment (Tremblay et al., 2010; Ransohoff and El Khoury, 2014; Schafer and Stevens, 2015; Miyamoto et al., 2016). Fractalkine receptor-deficient mice showed decreased neuron survival in layer V cortical neurons during the first postnatal week because of the reduced secretion of insulin-like growth factor from microglia (Ueno et al., 2013). Furthermore, microglia depletion by the *Cre-Lox* technique led to deficiencies in multiple learning tasks, as well as a significant reduction in learning-related synapse formation (Parkhurst et al., 2014). In addition, microglial-secreted BDNF could increase the activity of neuronal tropomyosin-related kinase receptor B, an important modulator in synaptic plasticity, prompting a crucial role of BDNF signaling in learning and memory-related synapse formation (Parkhurst et al., 2014). Microglia may communicate with neurons; changes in the physiological environment such as chronic stress and light deprivation can cause hyper-ramification of microglia and more frequent neuron–microglia contacts (Torres-Platas et al., 2014; Welberg, 2014; Yirmiya et al., 2015). Moreover, microglia act as scavengers in the adult CNS to remove damaged components, apoptotic cells, and misfolded proteins (Sierra et al., 2010). Once “danger” signals are detected in the surrounding environment, microglia rapidly change into a reactive phenotype, characterized by larger soma, and release various complement factors to facilitate neuron and tissue repair (Hanisch and Kettenmann, 2007; Graeber and Streit, 2010; Kettenmann et al., 2011).

### Functions of Non-parenchymal Macrophages

Non-parenchymal brain macrophages have been largely ignored, but recent studies have refocused on their relevant functions in the perivascular space, meninges, and choroid plexus (Herz et al., 2017). Perivascular macrophages are critical in BBB establishment (Mendes-Jorge et al., 2009; He et al., 2016), and blockage of colony stimulating factor 1 receptor signaling leads to a decreased coverage of pericytes in brain vessels (Yamamoto et al., 2017). Perivascular macrophages continuously retract and protract along blood vessels in the adult brain (Daneman, 2012; Goldmann et al., 2016), suggesting that they may play a role in immune surveillance. Moreover, they can protect against bacterial infection by recruiting circulating leukocytes (Polfliet et al., 2001). The study of meningeal macrophages has been limited; however, it was reported that meninges possess a “glymphatic” system, which guides the clearance of waste products such as β-amyloid in the CNS (Raper et al., 2016). However, whether meningeal macrophages instruct lymphangiogenesis in the “glymphatic” system, similar to their role in the peripheral immune system, remains to be ascertained (Gordon et al., 2011; Louveau et al., 2015). In addition, meningeal macrophages function as APCs, detecting cell debris and antigens to maintain immune homeostasis (Kivisakk et al., 2009). Choroid plexus macrophages are located near the microvilli of the choroid plexus (Liddelow, 2015; Marques et al., 2017), and their main function may support CSF release and flux (Goldmann et al., 2016). In conclusion, considerable fundamental details about non-parenchymal macrophages remain to be determined.

### Polarization of Microglia and Macrophages

Macrophages (including microglia) are often classified into M1 (inflammatory) and M2 (anti-inflammatory) phenotypes (Mikita et al., 2011; Liu et al., 2013). However, this simple classification does not reveal the full spectrum of macrophages under different stimulation conditions owing to their diversity and plasticity (Prinz and Priller, 2014). Transcriptome profiles have revealed at least nine different subtypes of human macrophages (Veremeyko et al., 2018). In this review, M1 and M2 represent the activation states of macrophages rather than specific cell subtypes.

Interferon-γ and lipopolysaccharide treatment can generate M1 cells, while IL-4, IL-10, or IL-13 are activators of M2 cells. M1 macrophages and microglia are characterized by an amoeboid shape, and they release pro-inflammatory cytokines, such as IL-6, IFN-γ, IL-23, TNF-α; inducible nitric oxide synthase; chemokines, including CCL 4, CCL5, and CCL8; and CXCL 2, CXCL4, and CXCL9 (Juhas et al., 2015; Kapellos and Iqbal, 2016). M1 macrophages and microglia are potent APCs, causing an adaptive immune response to clear foreign substances and abnormal proteins (Das et al., 2015). In contrast, M2 macrophages and microglia have smaller cell bodies and a branched structure (Ransohoff and Perry, 2009), and they express a variety of anti-inflammatory molecules, including IL-4, IL-10, IL-13, and transforming growth factor-β (David and Kroner, 2011). These contribute to immunoregulation by inhibiting inflammation, remodeling tissue, promoting angiogenesis, and clearing parasites (Banerjee et al., 2013). M2 microglia promote oligodendrocyte differentiation, and their depletion inhibits remyelination (Kotter et al., 2005; Miron et al., 2013). M2-polarized macrophages and microglia can be further subdivided into M2a, M2b, and M2c cells. These have different activation and functional mechanisms but have some biochemical overlap (Table 1; Murray et al., 2014). Increasing evidence suggests that M1 cells prefer an anaerobic environment, whereas oxygen consumption and oxidative phosphorylation benefit M2 macrophages (Shi et al., 2017; Wang et al., 2018).

**Table 1:** Polarization of macrophages and microglia (Murray et al., 2014).

Phenotype	Stimulation	Marker	Function
M1	IFN-γ, lipopolysaccharide	CD86, CD40, MHCII	T cell priming
M2a	IL-4, IL-13	CD206, FIZZ1, ARG1, YM1	Immunity against parasites, tissue repair, collagen formation
M2b	Immune complexes	MHC-II, CD86	Recruitment of regulatory T cells
M2c	IL-10, TGF-β1, glucocorticoids	CD163	Wound healing


In conclusion, microglia and non-parenchymal macrophages are independent immune populations in the CNS. Besides different cell origins, they have their own functions in CNS development, homeostasis, and tissue healing (Lopez-Atalaya et al., 2018). Under steady state, microglia are primarily responsible for supporting the development of the healthy brain, they shape and maintain the neuronal synaptic network, interact with neurons and produce beneficial components (Hagemeyer et al., 2017). And non-parenchymal macrophages strictly limits access of peripheral immune cells (T cells, B cells, and monocytes) reach the CNS parenchyma (Polfliet et al., 2001). Upon detecting “danger” signals, both microglia and non-parenchymal macrophages produce large amounts of chemokines and cytokines to promote leukocyte infiltration into the CNS parenchyma and function as APCs to reactivate T cells in the CNS (Galli et al., 2011; Krausgruber et al., 2011). Moreover, microglia phagocytose damaged cell debris, as well as unnecessary neurons and synapses resulting from defective differentiation and/or migration (Paolicelli et al., 2011; Lopez-Atalaya et al., 2018).

## Microglia and Macrophages in MS

After CNS injury and inflammation, while microglia situated in the parenchyma and macrophages in the choroid plexus, perivascular space, and the meninges are activated, monocytes from the blood cross the damaged BBB and become monocyte-derived macrophages in the CNS (Lassmann et al., 2001). These spatial differences may determine subtype-specific effector functions of these distinctive phagocytes in the diseased CNS. In this section, we compare and elaborate the functions of microglia and macrophages in the progression of MS and EAE.

### Distinguishing Microglia From Other Macrophages

A lack of specific markers that distinguish resident microglia from other macrophages in MS lesions hampers the accurate evaluation of their contributions to human brain pathology. Originally, the expression profiles of CD11b and CD45 allowed the discrimination of CD11b^+^CD45^med^ microglia from CD11b^+^CD45^hi^ monocyte-derived macrophages (Martin et al., 2017). However, such a classification remains controversial (Koeniger and Kuerten, 2017). Studies have suggested TMEM119 (Bennett et al., 2016; Satoh et al., 2016; Li et al., 2019), Sall1 (Buttgereit et al., 2016), Siglec-H (Konishi et al., 2017), and P2Y12 (Mildner et al., 2017) as more valuable markers for distinguishing microglia from other macrophages. For instance, TMEM119 is expressed exclusively on Iba1^+^CD68^+^ microglia and not on infiltrated Iba1^+^CD68^+^ macrophages within demyelinating lesions of MS (Satoh et al., 2016). However, TMEM119 expression is absent in immature microglia (Satoh et al., 2016). Sall1 expression is abundant in neuronal and glial progenitor cells during CNS development (Harrison et al., 2012; Buttgereit et al., 2016), and P2Y12 shows decreased expression in activated microglia (Amadio et al., 2014; Mildner et al., 2017). Almost all microglia in the CNS parenchyma expressed Siglec-H, from developmental to mature stages, and the expression was maintained in activated microglia after CNS injuries (Koso et al., 2018). In contrast, Siglec-H expression was largely absent from other myeloid cells in the CNS (Koso et al., 2018). Overall, these findings suggest that Siglec-H is a promising specific marker for the specific identification of microglia from CNS-associated macrophages. However, these results are all from mice, and human Siglec-L2 is only ∼42% homologous with Siglec-H (Zhang et al., 2006). Whether Siglec-L2 might be a marker for microglia warrants further investigation.

### Microglia

Pronounced microglial activation with high expression levels of pro-inflammatory genes can be found in lesion areas and normal-appearing white matter of MS patients (Lucchinetti et al., 2000; Singh et al., 2013; Zrzavy et al., 2017). These clusters of activated microglia are regarded as “pre-active lesions,” with an absence of leukocyte infiltration and demyelination; however, they may eventually develop into active demyelinating MS lesions (Singh et al., 2013). In contrast to healthy controls, the number of P2Y12^+^ homeostatic microglia is significantly reduced in the white matter of MS patients, and TMEM119^+^ microglia predominantly govern the edge of active lesions in MS (Zrzavy et al., 2017), indicating that microglial activation is related to disease development.

TSPO tracers have demonstrated inflammatory processes with microglia involvement in MS (Vas et al., 2008; Oh et al., 2011). These microglia are enriched with iron, express pro-inflammatory cytokines, and cause persistent tissue damage (Wisnieff et al., 2015). Although microglia are important in synaptic plasticity, this function is destroyed under pathological insults, which causes synaptic loss in MS and eventually, cognitive decline (Michailidou et al., 2015; Hong et al., 2016; Salter and Stevens, 2017; Di Filippo et al., 2018). Activated microglia in EAE mice may produce large amounts of TNF-α and thus induce excessive glutamate, leading to spontaneous and miniature excitatory post-synaptic currents (Centonze et al., 2009, 2010). Furthermore, astroglial ATP amplifies microglial signals and subsequently promotes glutamate release from astroglia, which directly inhibits synaptic transmission (Kettenmann et al., 2013). Classically activated neuroinflammatory microglia secrete TNF-α, and C1q, inducing A1 astrocyte dysfunction, thereby resulting in the dysfunction of both oligodendrocytes and neurons (Liddelow et al., 2017; Rothhammer et al., 2018). Importantly, mitochondrial disturbances have been demonstrated in modulating MS lesions, reactive oxygen species and reactive nitrogen species generated by the activated microglia also lead to intra-axonal mitochondrial injury (Balaban et al., 2005; Haile et al., 2017). Kinetics studies from EAE mice indicate that microglia are the first cell line to take up myelin antigens (Sosa et al., 2013), subsequently restimulating encephalitogenic T cells in the CNS through major histocompatibility complex (MHC) molecules and costimulatory molecules (Perry, 1998). Consistent with this, time-course studies in EAE mice showed that the appearance of inflammatory T cells in the CNS is consistent with the activation of CD11b^+^ microglia (Murphy et al., 2010). However, specific deletion of MHCII in microglia does not affect disease progression, suggesting that microglia are not important in reactivating efficient T cells in the CNS (Wolf et al., 2018). Microglia also secrete a series of pro-inflammatory cytokines such as IL-18, IL-6, and IL-1b and chemokines like CCL2 and CCL5 to aggravate both MS and EAE (Merson et al., 2010; Jiang et al., 2014). Moreover, activated microglia in white matter lesions may lead to greater brain atrophy in secondary progressive disease (Datta et al., 2017). In conclusion, microglia exert detrimental functions in MS and strongly damage myelin.

Microglial activation may also be beneficial for remyelination in MS due to the phagocytotic ability of microglia, as well as their secretion of neuroprotective molecules and anti-inflammatory cytokines (Du et al., 2017). Microglia-specific deletion of A20, the crucial protein in regulating microglial activation, makes microglia lose their ability to regulate neuronal synaptic function, thus exacerbating MS-like disease (Voet et al., 2018). Several receptors, including TREM2, complement receptor 3, and signal regulatory protein-α, invoke microglia to phagocytose myelin debris in lesions (Brendecke and Prinz, 2015). Microglia-specific ablation of TREM2 cannot upregulate genes associated with phagocytosis (Poliani et al., 2015), and the blockage of TREM2 results in increased demyelination in EAE (Piccio et al., 2007). Moreover, microglia secrete trypsinogen, insulin-like growth factor, and fibroblast growth factor during activation to promote neurogenesis (Pérez-Martín et al., 2010; Voss et al., 2012). Anti-inflammatory molecules from microglia also benefit remyelination in EAE, for example, IL-4 enhances oligodendrogenesis (Butovsky et al., 2006), and activin A drives oligodendrocyte differentiation (Miron et al., 2013). Interestingly, pro-inflammatory cytokine TNF has neuroprotective functions in EAE (Wolf et al., 2017), and deletion of microglial TNFR2 causing early disease onset, with increased leukocyte infiltration, demyelination and T cell activation in CNS (Gao et al., 2017). However, soluble TNF repair remyelination by suppressing clearance of myelin debris (Karamita et al., 2017). Other evidence indicated that microglia can prompt remyelination through P2X4R signaling, and that the blockade of P2X4R results in microglial activation to a pro-inflammatory phenotype and exacerbates the clinical symptoms of EAE mice (Zabala et al., 2018).

### Monocyte-Derived Macrophages

During the effector stage of EAE, Ly6C^hi^ monocytes rapidly infiltrate into the inflamed CNS with the help of matrix metalloproteinases (Ajami et al., 2011; Yamasaki et al., 2014). Monocytes then differentiate into macrophages, express MHC II and costimulatory molecules, and produce pro-inflammatory factors that actively contribute to the demyelination process (Jiang et al., 2014; Yamasaki et al., 2014). Consistently, the abundance of CCR1^+^/CCR5^+^ hematogenous monocytes has been found to appear in demyelination zones compared with the periplaque white matter of MS patients (Trapp et al., 1998; Trebst et al., 2001), and Kim1p staining shows abundant subcortical macrophage infiltration (Tobin et al., 2017).

The activated monocyte-derived macrophages is generally considered to be harmful in MS (Yamasaki et al., 2014). Infiltrating monocytes accumulate at the nodes of Ranvier during early stages of EAE and actively initiate the demyelination of axons (Yamasaki et al., 2014), similar to microglia, but they do not affect CNS-resident microglia (Ajami et al., 2011). Bone marrow chimeric mice show that TRPM2 expressed by CNS-infiltrating macrophages prompts the production of CXCL2, thus increasing neutrophil infiltration and contributing to the progression of EAE (Tsutsui et al., 2018). High F4/80^+^CD45^hi^ macrophage/T-cell ratios were found in EAE mice (McMahon et al., 2005), indicating that recruited macrophages participate in T-cell proliferation after myelin antigen stimulation. Macrophages also secrete large amounts of pro-inflammatory cytokines, such as IL-6, IL-12, TNF-α, IL-1, and IL-23, to aggravate the inflammatory response (Valentin-Torres et al., 2016). IL-1β expression has been found to be pivotal in the polarization of Th17 cells and therefore for EAE induction (Sutton et al., 2006). IL-6 has also been demonstrated to be critical for CNS autoimmunity, as IL-6-deficient mice demonstrated alleviated EAE (Samoilova et al., 1998). Macrophages also express high levels of CCR4 during the disease, and CCR4 deletion leading to reduced accumulation of macrophages in CNS and attenuated EAE symptoms (Forde et al., 2011). These findings suggest that macrophages are responsible for demyelination, but their direct functions on axons remain to be determined.

Other studies have also defined the neuron-supportive activities of Ly6C^hi^ monocytes in EAE. The activation of invariant natural killer T cells converts inflammatory Ly6C^hi^ monocytes to M2 macrophages in the CNS, with associated improvement in neurological impairment (Denney et al., 2012; Jiang et al., 2017). Recent studies have showed that TG2 mRNA levels are increased in monocytes derived from MS patients and correlates with anti-inflammatory cytokine expression, proposing a more anti-inflammatory monocytes status in MS (Sestito et al., 2017). Monocyte-derived macrophages can also promote CNS repair in the injured spinal cord by clearing myelin debris through scavenger receptors (GrandPré et al., 2000; Kotter et al., 2006). In contrast to microglia, macrophages express TREM-1 rather than TREM-2, and although this isoform plays a role in inflammatory responses (Colonna and Facchetti, 2003), the functions of TREM-1 still need to be further studied in a CNS autoimmunity context. In addition, monocyte-derived macrophages secrete trophic factors and anti-inflammatory factors to alleviate sympathetic neuron dysfunction (Hikawa and Takenaka, 1996; Miron et al., 2013). The transcription factor Nr4a1 represses the autocrine production of norepinephrine in macrophages, and myeloid cells lacking Nr4a1 lead to more leukocyte infiltration within the CNS and more severe EAE clinical symptoms (Shaked et al., 2015). Consistently, expression levels of Nr4a were down-regulated in peripheral blood mononuclear cells from MS patients, and Fingolimod treatment could recovery MS from Nr4a2 deficit (Montarolo et al., 2018). Besides Ly6C^hi^ monocytes, a small number of LyC6^lo^ monocytes also recruited in the injured CNS (Saederup et al., 2010). Although studies have pointed toward a reparative role of infiltrated Ly6C^lo^ monocytes in a spinal cord injury model (Arnold et al., 2007; Nahrendorf et al., 2007); however, more research is needed to determine whether Ly6C^lo^ monocytes do promote repair processes in the pathogenesis of EAE.

### Non-parenchymal Macrophages

Perivascular macrophages, typically CD163^+^HLADR^+^, are markedly increased in MS patients and EAE-affected animals (Zhang et al., 2011; Mammana et al., 2018). Substantial evidence indicates that perivascular macrophages have both supportive and neuroprotective roles in MS. Selective elimination of both perivascular and meningeal macrophages by treatment with clodronate-loaded liposomes reduces the progression of EAE in rats (Polfliet et al., 2002), suggesting that perivascular macrophages may act as competent APCs in EAE. EAE-related perivascular macrophages upregulate the expression of MHC II and can present peptides to myelin-specific T cells in the CNS, thereby accelerating disease progression (Perry, 1998; Fabriek et al., 2005; Greter et al., 2005; Vogel et al., 2013). Furthermore, perivascular macrophages express high levels of adhesion molecules such as VCAM-1 and ICAM-1 and chemokines like CCL2 and CCL3 (Hofmann et al., 2002; Vercellino et al., 2017), which may mediated by chondroitin sulfate proteoglycans (Stephenson et al., 2018), to promote leukocyte infiltration into the CNS. However, perivascular macrophages during the early stage of EAE are characterized as mannose receptor-positive M2 macrophages, produce leukemia inhibitory factors and neuroprotective factors, and secrete anti-inflammatory IL-6 and IL-1ra to protect glial and neuronal cells (Boven et al., 2006; Vanderlocht et al., 2006). Moreover, M2 perivascular macrophages can promote the differentiation of Th2 and Treg cells due to their anti-inflammatory characteristic (Weber et al., 2007).

An extensive number of meningeal immune cells in the CNS leads to a more severe clinical course of MS (Howell et al., 2011; Choi et al., 2012; Kilsdonk et al., 2017; Kolber et al., 2017; Bevan et al., 2018). In concordance, meningeal macrophages are equipped to present peptide antigens to T cells, triggering neuroinflammatory events (Bartholomäus et al., 2009; Kivisakk et al., 2009; Ransohoff and Cardona, 2010). In addition, meningeal macrophages can release inflammatory and toxic mediators or even invade the CNS parenchyma to sustain MS pathology (Maxwell et al., 1990; Garabedian et al., 2000; Reuter et al., 2001). In contrast, meningeal macrophages produce neurotrophic and anti-inflammatory factors to inhibit leukocyte activity and their infiltration into the CNS at particular disease stages (Bogie et al., 2014). Leptomeningeal macrophages increase the expression of transforming growth factor-β in glial cells and cortical neurons by releasing prostaglandin E2 during inflammation (Wu et al., 2007). However, other studies demonstrated that prostaglandin E2 could also exacerbate EAE (Lima et al., 2012), depending on the disease stage and microenvironment.

The choroid plexus, the site of CSF production, forms the interface between blood and the CNS (Wolburg and Paulus, 2010; Kaur et al., 2016). It is thus an important element in the communication between the vascular compartment and the CNS during inflammation (Breuer et al., 2018). The severity of MS coincides with enhanced levels of infiltrated leukocytes in the choroid plexus (Engelhardt et al., 2001; Vercellino et al., 2008; Mitchell et al., 2009), strongly indicating that inflammation at the choroid plexus contributes to the disease. Chemokines like CXCL9 and CXCL10 accumulate in large quantities in the CSF during MS progression (Edwards et al., 2013; Puthenparampil et al., 2017), but whether they are secreted by macrophages needs to be confirmed. Choroid plexus macrophages may release neurotoxic mediators and pro-inflammatory factors into the CSF during disease to impact perivascular and meningeal inflammation, as well as the activation and integrity of neurons and glial cells (Garabedian et al., 2000; Bragg et al., 2002a,b). However, remote brain choroid plexus can also recruit M2-like macrophages to an injured spinal cord, which might benefit myelin repair (Shechter et al., 2013).

### Foamy Macrophages

Ample evidence indicates that abundant foamy macrophages are present in MS lesions (Grajchen et al., 2018). After disease initiation, both resident microglia and recruited monocytes ingest and accumulate vast amounts of myelin-derived lipids and thereby acquire their distinctive morphology (Li et al., 1996; Bogie et al., 2017), but how foamy macrophages function in the process of MS is still poorly understood. MHC II and costimulatory molecules expressed on foamy macrophages may present myelin antigens to autoreactive T cells (de Vos et al., 2002; Boven et al., 2006; van Zwam et al., 2011), thereby driving epitope spread and MS progression or even initiation (Stys et al., 2012). In agreement with this, numerous studies have found M1-like foamy macrophages present within MS lesions (Bogie et al., 2014). Interestingly, yet another study showed that microglia show the M1 phenotype after the first 6 h of myelin uptake, and they soon convert to the M2 phenotype after prolonged exposure (Liu et al., 2006), indicating that the polarization of foamy macrophages can be reversed. Both *in vivo* studies and *in vitro* cultures demonstrated that foamy macrophages prefer to express anti-inflammatory molecules rather than secrete pro-inflammatory cytokines (van Zwam et al., 2011), indicating that myelin macrophages may resolve the inflammation in MS demyelination lesions. Consistent with this, researchers found that mouse myelin macrophages could inhibit IFN-γ release by Th1 cells and suppress EAE severity (Bogie et al., 2014). Collectively, these studies imply that besides being aggressors in MS pathogenesis, foamy macrophages can also destroy T cell-induced autoimmunity. However, more research is warranted to confirm the specific role of foamy macrophages in MS progression.

In fact, among numerous phagocytic cells, only microglia and infiltrating macrophages play a crucial role in MS progression. Both of them participate in demyelination and remyelination through antigen presentation, phagocytosis, and inflammatory mediator secretion (Shechter et al., 2009; Ruckh et al., 2012; Li and Barres, 2018); however, microglia alone could affect neuron cells directly through fractalkine-CX3CR1 signaling (Eyo et al., 2016). Although microglia and infiltrating macrophages are potent APCs in EAE, the expression levels of MHC II and costimulatory molecules on microglia are lower (Brendecke and Prinz, 2015), indicating that inflammation in the CNS is mainly activated by infiltrating macrophages. The roles of perivascular macrophages, as well as macrophages, in the meninges and choroid plexus in MS are not yet completely understood. In conclusion, whether these macrophages are unique phagocyte subsets in MS, and if so, the underlying mechanism thereof, needs to be determined.

## Therapeutic Perspectives

**FIGURE 2:**
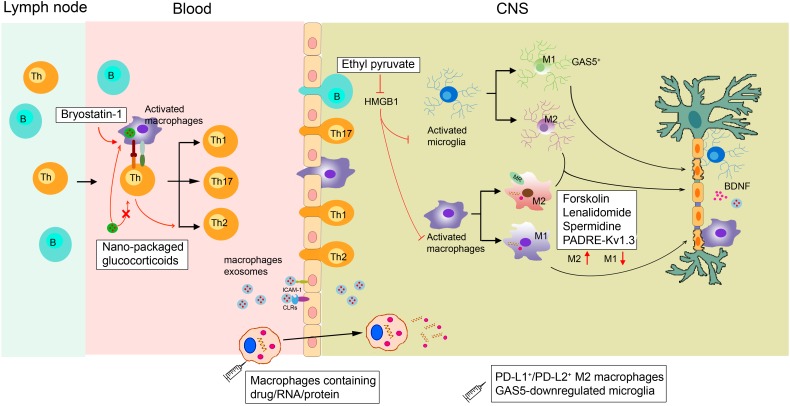
Proposed actions of disease-modifying multiple sclerosis therapies targeting macrophages. In the periphery, bryostatin-1 promotes the differentiation of lymphocytes into anti-inflammatory Th2 cells by acting on macrophages. Ethyl pyruvate prevents the activation of macrophages/microglia within the CNS. Lenalidomide, spermidine, forskolin, and the novel PADRE-Kv1.3 vaccine can promote macrophage/microglia M2 polarization. Transplanting GAS5-downregulated microglia into the lateral ventricles of EAE mice could promote remyelination. Exogenous supplementation of M2 macrophages such as PD-L1^+^/PD-L2^+^ M2 cells can also alleviate demyelination. Micro-based drug delivery systems for delivering GC to targeted macrophages have been widely used in EAE. As macrophages can easily migrate into the brain during MS, they can be used as cellular vehicles for the delivery of small drugs, RNAs, and proteins to the brain parenchyma. Macrophage exosomes could cross the BBB and deliver BDNF to recover injury. HMGB1, high-mobility group box 1; GC, glucocorticoids; MR, mineralocorticoid receptors; CLRs, C-type lectin receptors.

As discussed in the previous sections, any change in the physiological functions of macrophages and microglia can impact neuroinflammatory and neurodegenerative events. Therefore, selectively promoting macrophage polarization to the anti-inflammatory subtype is an attractive therapeutic option (Figure 2).

### MS Therapy by Regulating Macrophages

Although the crucial role of macrophages and microglia in neurodegenerative events has been proven, therapeutics solely targeting these phagocytes are still lacking. Currently, the clinical treatment of MS includes recombinant IFNβ-1a, glatiramer acetate, and natalizumab to mainly regulate T and B cells (Cheng et al., 2017). Studies have found that the neuroprotective effects of glatiramer acetate are mediated by activated M2 microglia (Ratchford et al., 2012). Furthermore, diverse new compounds that influence macrophage and microglial functions are undergoing non-clinical testing. These agents include ethyl pyruvate (Djedović et al., 2017), spermidine (Yang et al., 2016), bryostatin-1 (Kornberg et al., 2018), forskolin (Veremeyko et al., 2018), and lenalidomide (Weng et al., 2018). Ethyl pyruvate can reduce high-mobility group box 1 expression in activated ED1^+^ macrophages and Iba1^+^ microglia, thus inhibiting the activation of macrophages/microglia within the CNS to protect against EAE (Djedović et al., 2017). In addition, forskolin has been found to alleviate EAE by suppressing the expression of CD86 while enhancing the expression of ARG1 in macrophages (Veremeyko et al., 2018). Bryostatin-1 provides marked benefits in mice with EAE by acting on APCs, including macrophages, to promote the differentiation of lymphocytes into Th2 cells (Kornberg et al., 2018).

The novel PADRE-Kv1.3 vaccine has been applied to treat EAE. After vaccination, infiltrated microglia/macrophages significantly shift toward the M2 subtype in the CNS (Fan et al., 2018). Mineralocorticoid receptors expressed on macrophages promote their polarization toward M2 cells, and mice selectively lacking mineralocorticoid receptors in their myeloid cells show diminished clinical symptoms of EAE (Montes-Cobos et al., 2017b). Transferring M2 macrophages to eliminate EAE has been widely applied. The long non-coding RNA GAS5, a suppresser of microglial M2 polarization, is expressed at a high level within microglia in MS patients. Transplanting GAS5-downregulated microglia into the lateral ventricles of EAE mice could promote remyelination, similar to a lysolecithin-induced demyelination model (Sun et al., 2017). In addition, Ly6C^+^ monocytes could give rise to PD-L1^+^/PD-L2^+^ M2 cells, and transferring PD-L1^+^/PD-L2^+^ M2 macrophages might reduce the incidence of EAE (Terrazas et al., 2017).

### Macrophage-Targeted Therapeutics via Micro-Based Drug Delivery Systems in Neurodegenerative Diseases

Because most drugs do not effectively reach macrophages at therapeutic levels, micro-based drug delivery systems for targeting macrophages have been widely used in treating CNS diseases. For example, glucocorticoids are used extensively to treat acute relapses in MS patients (Pato-Pato et al., 2003; Cohen et al., 2009), but their therapeutic effect is accompanied by serious side effects due to their broad spectrum of immunosuppressive actions (Baschant and Tuckermann, 2010). Interestingly, *in vivo*, glucocorticoids packaged with specific nanoparticles vs. free glucocorticoids are preferentially taken up by phagocytic cells, including macrophages, rather than T cells. This approach allows their therapeutic efficacy to be retained in EAE mice (Montes-Cobos et al., 2017a). Other research showed that infiltrated CD163^+^ macrophages in brains from a Parkinson’s disease model modulate local microglia to promote neuroprotection. Specifically designed liposomes can load dexamethasone solely to CD163^+^ macrophages. This modification leads to decreased dopaminergic cell death and better motor performance (Tentillier et al., 2016). However, we cannot ignore that several nanoparticles themselves also induce macrophage polarization toward various phenotypes and modulate macrophage reprogramming (Miao et al., 2017).

### Macrophages as Drug Carriers for MS Therapy

Many CNS diseases are thought to be untreatable because drugs rarely pass through the BBB at therapeutic levels (Fischbach et al., 2013). Chemokine gradients secreted from the CNS parenchyma can induce macrophage migration to the brain, making macrophages potential carriers for drug/nano formulations across the BBB to reach target sites (Ye et al., 2018). Macrophages transporting superparamagnetic iron oxide nanoparticles and exogenous genes were injected intravenously into a lipopolysaccharide-induced acute neuroinflammatory mouse model. The differentiated macrophages demonstrated an excellent ability to spread into the brain, and the number of transported macrophages positively correlated with the number of intravenous cells (Tong et al., 2016). Moreover, macrophage exosomes can interact with brain microvessel endothelial cells that comprise the BBB by binding to carbohydrate-binding C-type lectin receptors. BBB cells can absorb more macrophage exosomes when inflammation occurs due to the upregulated secretion of ICAM-1. After intravenous administration, macrophage exosomes have been shown to cross the BBB and deliver a cargo protein, BDNF, in the presence of CNS inflammation (Yuan et al., 2017). Overall, these results support the concept of using monocytes/macrophages or macrophage exosomes as cellular vehicles for the delivery of small drugs, RNAs, and proteins to the brain parenchyma.

## Perspective

It is becoming increasingly clear that microglia and macrophages play critical roles in healthy, inflamed, injured, and recovering CNS due to their dual natures. We have summarized the origin, subtypes, and functions of these phagocytic cells in MS. Therapeutic interventions that block the pro-inflammatory effects of macrophages/microglia during disease progression, while preserving their anti-inflammatory functions on EAE, have achieved great success. However, as infiltrating inflammatory monocytes and microglia contribute differentially to EAE pathophysiology, strategies that allow the differential manipulation of CNS-resident microglia and infiltrating macrophages to optimally target different mechanisms operating in MS need to be developed. Moreover, extensive additional research is needed before utilizing the physiology of microglia and macrophage subsets for MS therapy.

## Author Contributions

QW and QH conceived the review article and made the corrections in the manuscript. BY provided some critical comments. JyW wrote the manuscript. JjW and JcW collected the related research articles.

## Conflict of Interest Statement

The authors declare that the research was conducted in the absence of any commercial or financial relationships that could be construed as a potential conflict of interest.

## Abbreviations

APCs: antigen-presenting cells
BBB: blood–brain barrier
BDNF: brain-derived neurotrophic factor
CCL: C-C motif chemokine ligands
CNS: central nervous system
CSF: cerebrospinal fluid
CXCL: C-X-C motif chemokine ligands
EAE: experimental autoimmune encephalomyelitis
IFN: interferon
IL: interleukin
MHC: major histocompatibility complex
MS: multiple sclerosis
TNF: tumor necrosis factor
